# Incidence and Causes of Tubal Occlusion in Infertility: A Retrospective Cohort Study

**DOI:** 10.3390/jcm13133961

**Published:** 2024-07-06

**Authors:** Daniel Mayrhofer, Iris Holzer, Judith Aschauer, Clara Selzer, John Preston Parry, Johannes Ott

**Affiliations:** 1 Clinical Division of Gynecologic Endocrinology and Reproductive Medicine, Medical University of Vienna, 1090 Vienna, Austria; daniel.mayrhofer@meduniwien.ac.at (D.M.);; 2 Department of Obstetrics and Gynecology, Louisiana State University Health Shreveport, Shreveport, LA 71103, USA; 3 Parryscope and Positive Steps Fertility, Madison, MS 39110, USA

**Keywords:** fallopian tubes, patency testing, fertility, fallopian tube diseases, uterine leiomyomas, male infertility

## Abstract

**Background and Objectives**: Fallopian tubal pathology is a primary risk factor for female infertility, with simple proximal disease and proximal disease extending more distally being more common than pure distal occlusion. Proximal tubal occlusion is often attributed to ascending events, such as pelvic inflammatory disease. Conversely, while distal occlusion can also be attributable to ascending pelvic inflammatory disease, it can also have a pelvic origin, such as through endometriosis and ruptured appendicitis. The aim of this study was to identify certain causes of infertility and their association with tubal occlusion. The focus was on the location of tubal occlusion, uni- versus bilateral occlusion, and other causes of infertility, including male factors. **Methods**: In a retrospective study cohort study, 373 women aged between 18 and 40 years, treated from 1 January 2017 to 31 December 2022, were included. Fallopian tube patency was tested using either hysterosalpingography, hysterosalpingo-contrast sonography, or laparoscopic chromopertubation. **Results**: In total, 95 of 373 women (25.5%) revealed at least one occluded tube, with unilateral occlusion being more common than bilateral occlusion (60/95, 63.2% vs. 35/95, 36.8%). The majority of tubal occlusions occurred proximally (86.2%). According to the adjusted multivariate regression models, the presence of hydrosalpinx (odds ratio, OR, 13.323, 95% confidence interval, CI: 2.679–66.253, *p* = 0.002), myomas (OR 2.108, 95%CI: 1.008–4.409; *p* = 0.048), and an abnormal sperm test result of the male partner (OR 2.105, 95%CI: 1.156–3.833; *p* = 0.015) were statistically significant associated factors for tubal occlusion. **Conclusions**: Fallopian tube patency testing is still of major relevance in fertility evaluation. The presence of uterine myomas, hydrosalpinges, and a male factor significantly increase the risk.

## 1. Introduction

Infertility is defined as the inability to establish a clinical pregnancy after 12 months of regular unprotected intercourse with the same partner and affects up to 12% of couples of reproductive age [1]. Although approximately 15–30% of infertile couples are without a clear cause, the most commonly identified reasons for unwanted childlessness are ovulatory dysfunction, male factor infertility, endometriosis, diminished ovarian reserve, uterine and cervical factors, and tubal disease [2,3]. According to the joint committee opinion by the American College of Obstetricians and Gynecologists’ Committee on Gynecologic Practice and the American Society for Reproductive Medicine regarding infertility workup, basic infertility evaluation consists of patient history, physical examination, hormonal status, semen analysis, and uterine and tubal evaluation, such as transvaginal ultrasound and hysterosalpingography. In addition, further diagnostic steps and more invasive procedures should not be performed routinely, such as endometrial biopsy and laparoscopy, for unexplained infertility [4]. The gold standard for tubal patency testing is laparoscopy with chromopertubation, as tubal patency and pelvic anatomy can be assessed simultaneously [5,6]. Combined with hysteroscopy, this procedure allows the evaluation of the uterine cavity to identify anatomical, endometrial, and congenital abnormalities [7]. Accordingly, diagnostic laparoscopy may be indicated in the setting of intra-abdominal adhesions, myomas, or hydrosalpinges either when there is an indeterminate answer with a screening test or when evaluation is also intended to overlap with treatment [8,9]. Although hysterosalpingography (HSG) and hysterosalpingo-contrast sonography (HyCoSy) lack information on pelvic pathology, they have been considered the first choice in women with a low risk for tubal pathology, as they are less invasive and more cost-effective, show comparable diagnostic accuracy compared to chromopertubation, and can also be performed as office procedures [5,6]. A major disadvantage of both tests is that they are highly operator-dependent. HSG exposes women to ionizing radiation, does not provide information on ovarian morphology, and is generally considered more painful. Thus, HyCoSy has become increasingly popular and has widely replaced HSG for tubal infertility evaluation [5,7,10]. HSG and HyCoSy have comparable diagnostic accuracy compared to laparoscopic chromopertubation, with a sensitivity ranging from 85 to 95% and 86 to 94%, and a specificity from 89 to 93% and 92 to 94%, respectively [5,10,11]. The procedures are described in more detail in the Materials and Methods section below. Tubal pathology can account for up to 35% of infertility, with 10–25% of women having occlusion at the proximal end [12]. While recent theories hypothesize that proximal occlusion might be a result of tubal spasms, incorrect introduction of dye or distention medium during patency testing, or retained intraluminal debris, the most common cause of true distal occlusion is pelvic inflammatory disease [13,14]. Some studies find proximal tubal occlusions to be more prevalent than distal ones [8,9], with one study showing a twenty-fold higher prevalence of proximal occlusion in women with chronic endometritis [15]. The higher frequency of proximal tubal disease also coincides with our clinical observations. One theoretical explanation for this gap between historical and current observations is that improved medical diagnostics and treatment of pelvic inflammatory disease may have shifted the nature of upper genital infection so that the disease is less likely to ascend beyond the uterus and into the peritoneal cavity.

To the best of our knowledge, there is no recent literature about the current incidence of tubal occlusion in women with infertility. To evaluate this issue and to clarify whether factors associated with infertility would be associated with tubal occlusion, we performed a retrospective cohort study in women who underwent a full evaluation for infertility at our department. Accordingly, this study aims to determine the incidence of tubal occlusion and identify associated factors in women undergoing infertility. The focus was on the location of tubal occlusion, uni- versus bilateral occlusion, and other causes of infertility, including male factors.

## 2. Materials and Methods

### 2.1. Patient Population

In a retrospective data set, all consecutive women aged 18–40 years who underwent evaluation of tubal patency for primary or secondary infertility at the Clinical Division of Gynecologic Endocrinology and Reproductive Medicine of the Medical University of Vienna, Austria, from 1 January 2017 to 31 December 2022, were included (*n* = 373). The upper cut-off of 40 years was chosen based on the age-dependent decline in female fertility, with a loss of fertility of about 50% at this age. All included women had at least one fallopian tube. To minimize the risk of selection bias, women with previous tubal reconstruction surgery were excluded. One woman had a unicornuate uterus and, thus, only one tube. No additional exclusion criteria were applied. This study was approved by the Institutional Review Board of the Medical University of Vienna (IRB number 1836/2022). Due to the retrospective study design, obtaining informed consent was not necessary.

### 2.2. Parameters Analyzed

Data acquisition was conducted using AKIM Software (version 7, SAP Software Solutions Austria, Vienna, Austria; SAP-based patient management system at the Medical University of Vienna). The main outcome parameter was fallopian tube patency diagnosed using either HSG, HyCoSy, or laparoscopic chromopertubation, which were performed as published previously [8,16]. Laparoscopic chromopertubation was conducted under general anesthesia and performed or supervised by experienced experts in infertility surgery. Therefore, a Spackman uterine manipulator with a clamp fixation and rubber cone (18 mm diameter) (reference number 1264; WISAP^®^ Medical Technology GmbH Brunnthal/Hofolding, Brunnthal, Germany) was located through the cervix, with the tip placed one centimeter from the uterine fundus. For chromopertubation, we used a syringe with 50 mL indigo carmine blue dye dilute solution (Amino AG, Gebenstorf, Switzerland) to visualize the open fallopian tubes. HSG and HyCoSy were performed and reported by experienced experts according to international standards [17,18]. Tubal patency testing was performed within the three months after the first consultation. Distal tubal occlusion was diagnosed when passage of distention medium/dye beyond the ampullary segment of the tube but not the fimbria was observed, whereas proximal occlusion was identified as the absence of visible passage of distention medium/dye beyond the isthmus of the fallopian tube [8].

The focus was also on other fertility-specific parameters. Polycystic ovarian syndrome (PCOS) was defined according to the revised Rotterdam criteria. According to these, at least two of the following criteria were to be fulfilled to diagnose PCOS: (i) clinical and/or biochemical hyperandrogenism, (ii) oligo- and/or anovultion, and (iii) polycystic ovaries visualized with ultrasound with at least 12 follicles with a diameter of 2–9 mm per ovary, as well as 17-hydroxy progesterone levels < 2 ng/mL, which permitted the identification of non-classical congenital adrenal hyperplasia where present [19,20]. The diagnosis of endometriosis, myomas, and endometrial polyps was established when confirmed hysteroscopically/laparoscopically and histologically. If hydrosalpinx was suspected during transvaginal ultrasound examination, laparoscopic evaluation was recommended, and in case of surgery, patency testing via chromopertubation was performed. In accordance with the recommendations of the Endocrine Society, functional hypothalamic amenorrhea (FHA) is defined by (i) amenorrhea or a menstrual cycle length persistently exceeding 45 days, (ii) a history of excessive weight loss/intense physical activity/psychological stress, and (iii) the presence of hypogonadotropic hypogonadism [21]. Premature ovarian insufficiency (POI), defined as the cessation of ovarian function before the expected onset of menopause, is characterized by (i) oligo/amenorrhea for at least four months, and (ii) elevated follicle-stimulating hormone (FSH) levels > 25IU/l on two occasions more than four weeks apart [22]. Sperm evaluation was performed according to the World Health Organization’s (WHO) laboratory manual for the examination and processing of human semen. A sperm test is considered abnormal with either (i) a sperm count below 16 million/mL, (ii) a total sperm count ≤ 39 million, (iii) ≤32% progressively sperm motility, (iv) normal morphology in <4%, (v) ≥50% dead sperm, and/or (vi) a total volume < 2 mL or >5 mL [23]. Additional information collected included the diagnosis of unexplained infertility, defined as the absence of common causes for infertility, such as the presence of PCOS, FHA, POI, endometriosis, endometrial polyps, male factors, or fallopian tube pathology (occlusion, hydrosalpinx). In addition, the following standard characteristics were also evaluated: age, body mass index (BMI), number of tubes, type of infertility (primary/secondary), gynecologic/obstetric history, and previous abdominal surgery.

### 2.3. Statistical Analysis

Categorical variables are presented as numbers (frequencies) and numerical variables as medians (interquartile range, IQR). Differences between groups were tested using the chi-square test for categorical parameters or Fisher’s exact test when at least one cell had an expected frequency < 10%. Factors associated with the presence of tubal occlusion, a binary categorical parameter, were tested using univariate followed by a multivariate binary logistic regression model. This was done, since the number of variables tested was too large to include all parameters in multivariable models together. For these models, odds ratios (ORs) with their 95% confidence intervals (95% CIs) and *p*-values are provided. Statistical analysis was performed using SPSS 29.0 for Windows (IBM Corp., Armonk, NY, USA, 1989–2023).

## 3. Results

### 3.1. Basic Patient Characteristics

The median patient age was 33 years (IQR 29–36) and the median BMI was 23.9 kg/m^2^ (IQR 21.1–28.0) at the time of evaluation of tubal patency. Women suffered from primary infertility or secondary infertility in 206 (55.2%) and 167 (44.8%) cases, respectively. Additionally, 66 (17.7%), 18 (4.8%), and 25 (6.7%) women had experienced previous first-trimester miscarriage, extrauterine pregnancy, and termination of pregnancy. Eleven women had only one tube remaining, 10 of which were attributable to unilateral salpingectomy, and one woman had a unicornuate uterus. General patient characteristics, including history of previously performed abdominal surgeries, are presented in Table 1.

### 3.2. Main Findings about Tubal Patency Testing

**Figure 1:**
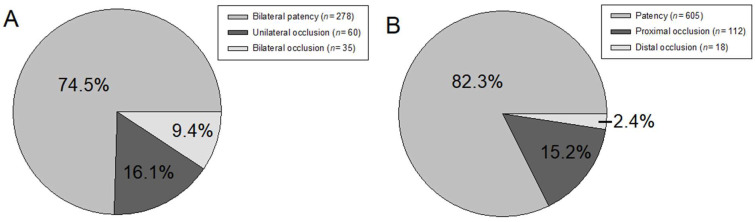
Overview of patency findings. The number of patients with bilateral patency, unilateral occlusion, or bilateral occlusion is provided in (**A**). The number of patency and proximal or distal occlusion per fallopian tube is shown in (**B**).

An overview of tubal patency is provided in Figure 1. About a quarter of all 373 women revealed any kind of tubal occlusion (95, 25.5%), with unilateral being more common than bilateral occlusion among these women (60/95, 63.2% vs. 35/95, 36.8%). In all of the 373 women, a total of 735 fallopian tubes were assessed. A hundred and thirty (17.7%) tubes were occluded, with 112/130 (86.2%) and 18/130 (13.3%) occlusions located proximally and distally, respectively. When the method for tubal patency testing was compared to the results (Table 2), HyCoSy revealed the highest rates of unilateral and bilateral occlusion per patient (*p* = 0.019) and was the most likely to identify a tube as occluded (*p* < 0.001).

### 3.3. Other Causes for Infertility and Their Association with Tubal Occlusion

Several reasons for infertility were present independent of tubal patency. With 168 (45.0%) cases, the male factor turned out to be the most common identifiable cause for subfertility within our population, followed by PCOS, presence of myomas, and endometriosis occurring in 72 (19.3%), 58 (15.5%), and 54 (14.5%) women, respectively. Without counting the results of tubal patency testing, the causes of infertility remained “otherwise unexplained” in 68 (18.2%) women. Table 3 provides an overview of tubal occlusion in accordance with the other causes for infertility.

In the last step, possible factors associated with the presence of any kind of tubal occlusion (unilateral or bilateral occlusion) were tested (Table 4). All factors that reached statistical significance in the univariate model also did so in the multivariate model apart from age (univariable model, OR 1.059, *p* = 0.020; multivariable model, adjusted OR 1.055, *p* = 0.051). In detail, the following parameters were associated with an increased risk for the presence of unilateral or bilateral occlusion: presence of hydrosalpinx (OR 13.323, 95% CI: 2.679–66.253; *p* = 0.002), myomas (OR 2.108, 95% CI: 1.008–4.409; *p* = 0.048), and an abnormal sperm test result of the male partner (OR 2.105, 95% CI: 1.156–3.833; *p* = 0.015). In contrast, if no other cause for infertility was found in the couple (“otherwise unexplained infertility”) prior to tubal patency assessment, the risk for any tubal occlusion was decreased (OR 0.204, 95% CI: 0.057–0.733, *p* = 0.015). BMI, nicotine consumption, parity, previous abdominal surgeries, and gynecological–endocrinological disorders were not predictive (*p* > 0.05).

**Figure 2:**
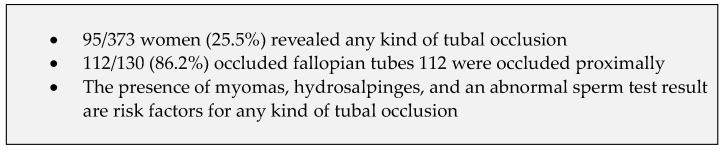
Summary of key findings.

Figure 2 shows a summary of our key findings.

## 4. Discussion

In our patient population of 373 consecutive unselected couples who had been referred for infertility, about a quarter of all women were found to have unilateral or bilateral tubal occlusion. About 18% of all fallopian tubes were occluded.

For context, it is well known that women in developed countries tend to delay childbirth [24] and that infertility increases with advancing age [25]. In our patient population, the median age was 33 years at initial presentation, with an IQR from 29 to 36, which might seem high but is comparable to other recent studies about fallopian tube patency in infertile women [10,16,26]. Notably, in 65 women (17.4%), unexplained infertility was found after the complete evaluation with tubal patency assessment. This rate fits into the reported range of 10–30% [27].

The overall rate of women affected by tubal occlusion in our population was about 25%. In older studies, it has been estimated that tubal pathology would account for up 14% to up to 35% of infertility [12,28]. Although our findings seem to fit into this range, it needs to be acknowledged that only 35/373 women demonstrated bilateral occlusion (9.4%). However, unilateral occlusion can still raise concerns for bilateral tubal disease. Not only does unilateral occlusion lower conception rates, but the technically patent tube can still have intraluminal damage hindering ciliary function. Accordingly, unilateral disease can be an important clue to etiology in women who thought their fertility to be “unexplained”. Of course, in the case of bilateral tubal occlusion, in vitro fertilization (IVF) instead of other assisted reproductive technologies (ART), e.g., intrauterine insemination (IUI), is inevitable. In the presence of unilateral tubal blockage, the further procedure depends on the site of tubal occlusion. A recent meta-analysis by Tan et al. demonstrated that cumulative pregnancy rates (CPRs) after intrauterine insemination (IUI) were similar between women with proximal unilateral tubal occlusion and women with unexplained infertility (odds ratio (OR) = 1.10, 95% confidence interval (CI) = 0.75–1.62), but patients with distal unilateral occlusion had significantly lower CPR (OR = 0.49, 95% CI = 0.25–0.97) [29]. Consequently, for women with distal unilateral tubal occlusion, a referral to specialists for assisted reproduction might be warranted earlier than expected.

Multiple pathophysiological mechanisms for proximal tubal blockage have been described. Most authors agree that the main causes consist of tubal spasms, past pelvic inflammatory diseases, hormonal and anatomical factors, impaired cilial activity, endometrial lesions/polyps, and the presence of amorphous material inside the tubes, causing obstruction [30,31]. The surprisingly high number of proximal tubal occlusions should be taken into account in fertility counseling. As mentioned above, women with proximal unilateral occlusion have better CPR with IUI than women with distal blockage. Thus, depending on the financial and social situation of the patients, less invasive procedures (e.g., IUI) can be recommended first and before directly referring to IVF.

Moreover, our system is one with universal healthcare, which is associated with a lower rate of sexually transmitted infections, so tubal disease may be even more meaningful in settings with more limited medical access.

Notably, there is a lack of recent studies about the prevalence of tubal occlusion in infertile women in the Western world. The most recent data were derived from our study group and included women with laparoscopic chromopertubation only [6,7]. It seems reasonable that the rates of tubal occlusion were similar to those in the present study. However, this underlines the importance of our up-to-date findings as well as the need for more studies on tubal patency epidemiology.

Additionally, in contrast to older findings [12], the vast majority of non-patent tubes showed a proximal occlusion (86.2%). One might hypothesize that pelvic inflammatory disease has become less prevalent [32] where earlier testing and treatment has led to less extensive spread. This may also apply to peritoneal pathology, such as ruptured appendicitis and moderate to severe endometriosis, where earlier detection and treatment could result in less peritubal disease and fimbrial agglutination. Finally, if classical sources for distal tubal damage are being more recognized, this increases the relevance of pathology from more cryptic sources, such as endometritis. Ultimately, our findings clearly demonstrate the need for fallopian tube patency evaluation in women of infertile couples due to the high prevalence of abnormal findings, even in a population with high access to care. Also of interest is the high rate of tubal disease in women with concurrent male factor subfertility. Although it is possible that these could overlap through infections leading to epididymitis, a simpler explanation would be a two-hit hypothesis. This would be where normal sperm might be able to overcome issues with a woman having a single functional tube, but if counts are low, decreasing the odds of sperm and egg meeting, and if one tube is also dysfunctional, these problems are more likely to be clinically noticeable.

It was surprising that several factors were not associated with fallopian tube occlusion. For example, previous extrauterine pregnancy without salpingectomy was not associated in a statistically significant fashion; however, this was likely an issue related to small sample size, affecting only 4.8% (18/373) of women. The same was true for the association of endometriosis with tubal occlusion [19,33]. Unfortunately, for endometriosis, the database did not offer details about staging. It is likely that the majority of these women were identified with only minimal to mild endometriosis, which is far less associated with tubal disease than advanced endometriosis [33].

Regarding study limitations, due to the retrospective study design, causal relationships can only be assumed with caution. Despite the fact that we included nearly 400 consecutive patients, the study population could still be larger to detect minor influences, such as the impact of age on the presence of tubal occlusion, which did not reach statistical significance in our multivariable model (Table 4). The lack of some demographic information and socioeconomic status in hindsight should have been occluded. First and foremost, the lack of information about previous pelvic inflammatory disease is a major drawback. Additionally, differences in the methods of assessing patency might have led to detection bias. The fact that occlusion rates differed between the different diagnostic tools used suggests either potential inconsistency in diagnostic accuracy or a selection bias, which must be seen as a study limitation. HyCoSy and chromopertubation showed a higher rate of tubal occlusion. Of note, HyCoSy is not covered by the social insurance in Austria and was performed at our department without additional cost for women, typically with suspicion of higher risk conditions, such as endometriosis, hydrosalpinges, and multiple myomas. Similarly, chromopertubation was done only for women with risk high enough to warrant surgery. Moreover, there could have been an additional selection bias due to the fact that not all infertile women are referred to a tertiary care center such as the Medical University of Vienna. One could hypothesize that mainly patients considered to have more severe problems might have been included in this study. Last but not least, our findings are based on a single medical facility in Austria and therefore might not be applicable to other populations with different demographic and health characteristics.

Ultimately, though this and all studies have opportunity for refinement, the preponderance of literature on the prevalence and location of tubal disease is decades old, and therefore may not reflect modern screening, therapies, and access to care [12,28]. Moreover, by including all women with subfertility undergoing tubal assessment at our institution over a six-year period, this reduces the risk for enrollment bias and enhances generalizability.

## 5. Conclusions

Despite advances in diagnosing and treating pelvic pathology over the last few decades, fallopian tubal occlusion remains high in infertile women, with over 25% of women participating having unilateral or bilateral fallopian tubal occlusion. Women with subfertility should undergo tubal patency testing early during infertility evaluation in order to not delay further treatment, particularly women with hydrosalpinges and myomas, as those were found to have the highest risk for occlusion. Although our results provide further insight into fallopian tube pathology, prospective data with a focus on demographics, history of pelvic inflammatory disease, the association of tubal occlusion and the stage of endometriosis, and underlying mechanisms of proximal tubal occlusion are needed.

## Figures and Tables

**Table 1:** General patient characteristics.

Number of women		373
Age (years) *		33 (29; 36)
BMI (kg/m^2^) *		23.9 (21.1; 28.0)
Number of tubes		735
Women with one tube ^#^		11 (2.9)
Secondary infertility ^#^		167 (44.8)
Gravidity ^#^	0	208 (55.8)
	1	94 (25.2)
	≥2	71 (19.0)
Parity ^#^	0	270 (72.4)
	1	75 (20.1)
	≥2	28 (7.5)
Previous first-trimester miscarriage ^#^		66 (17.7)
Previous extrauterine pregnancy ^#^		18 (4.8)
Previous termination of pregnancy ^#^		25 (6.7)
Previous intrauterine fetal death ^#^		3 (0.8)
Current smoking ^#^		45 (12.1)
Previous abdominal surgery ^#^	Cesarean section	41 (11.0)
	Curettage	49 (13.1)
	Laparoscopy: unilateral salpingectomy	10 (2.7)
	Laparoscopy: ovarian cyst removal	25 (6.7)
	Laparoscopy: endometriosis	14 (3.8%)
	Laparoscopy or laparotomy: myoma enucleation	9 (2.4)
	Laparoscopy: ovarian drilling	9 (2.4)
	Diagnostic laparoscopy	36 (9.7)
	Appendectomy	33 (8.8)
	Other intraperitoneal procedures	14 (3.8)
Previous hysteroscopic myoma resection		4 (1.1)

Date are presented as * median (IQR) or ^#^ number (%); One woman with a unicornuate uterus and only one tube!

**Table 2:** Results of tubal patency testing according to the method used.

	Number of Evaluated Women	Unilateral Occlusion	Bilateral Occlusion	*p*	Number of Evaluated Tubes	Number of Occluded Tubes	*p*
Hysterosalpingography	186	23 (12.4)	12 (6.5)	0.019	368	47 (12.8)	<0.001
HyCoSy	56	14 (25.0)	8 (14.3)		109	30 (27.5)	
Chromopertubation	131	23 (17.6)	15 (11.5)		258	53 (20.5)	

**Table 3:** Factors for subfertility independent of tubal patency testing.

	Number of Women (n, %)	Unilateral Occlusion	Bilateral Occlusion	Total Number of Tubes	Occluded Tubes
Previously known or newly diagnosed endometriosis *	54 (14.5)	9 (16.7)	6 (11.1)	108	21 (19.4) ^§^
PCOS *	72 (19.3)	9 (12.5)	3 (4.2)	143	15 (10.5) ^§^
Hypogonadotropic hypogonadism *	10 (2.7)	1 (10.0)	0	20	1 (5.0) ^§^
Premature ovarian insufficiency *	5 (1.3)	1 (20.0)	0	10	1 (10.0) ^§^
Myoma *	58 (15.5)	14 (24.1)	9 (15.5)	113	32 (28.3) ^§^
Endometrial polyp *	25 (6.7)	3 (12.0)	3 (12.0)	49	9 (18.4) ^§^
Male factor *	168 (45.0)	38 (22.6)	20 (11.9)	335	78 (23.3) ^§^
One tube missing *	11 (2.9)	4 (36.4)	-	11	4 (36.4) ^§^
Otherwise unexplained infertility ^#^	68 (18.2)	1 (1.5)	2 (2.9)	136	5 (3.7) ^§^

Data are presented as numbers (%); * multiple mentions are possible; ^#^ this excludes all women with above-mentioned * causes; ^§^ refers to the total number of tubes.

**Table 4:** Univariable followed by a multivariable binary regression model for the prediction of any kind of tubal occlusion (unilateral and/or bilateral).

		Women with Uni- or Bilateral Occlusion (*n* = 95)	Women with Bilateral Patency (*n* = 278)	OR (95%CI)	*p*	Adj. OR (95%CI)	Adj. *p*
Age (years) *		34 (30; 38)	32 (29; 36)	1.059 (1.009; 1.112)	0.020	1.055 (1.000; 1.113)	0.051
BMI (kg/m^2^) *		24.5 (21.3; 28.4)	23.4 (21.0; 27.9)	1.019 (0.976; 1.064)	0.392	-	-
Smoking ^#^		13 (13.7)	32 (11.5)	1.219 (0.610; 2.433)	0.575	-	-
Secondary infertility ^#^		47 (49.5)	114 (41.0)	1.388 (0.869; 2.215)	0.170	-	-
Parity	0	69 (72.6)	201 (72.3)	reference	0.892	-	-
	1	18 (18.9)	57 (20.5)	0.920 (0.507; 1.670)		-	
	≥2	8 (8.4)	20 (7.2)	1.165 (0.491; 2.766)		-	
Previous extrauterine pregnancy without salpingectomy ^#^		4 (4.2)	6 (2.2)	2.519 (0.751; 8.450)	0.135	-	-
Previous salpingectomy ^#^		5 (5.3)	6 (2.2)	1.993 (0.550; 7.219)	0.294	-	-
Previous Cesarean section ^#^		13 (13.7)	28 (10.1)	1.416 (0.700; 2.860)	0.333	-	-
Previous other intraperitoneal surgery ^#^		36 (37.9)	77 (27.7)	1.593 (0.975; 2.602)	0.063	-	-
Previously known or newly diagnosed endometriosis ^#^		15 (15.8)	39 (14.0)	1.149 (0.602; 2.195)	0.674	-	-
PCOS ^#^		12 (12.6)	60 (21.6)	0.525 (0.269; 1.026)	0.059	-	-
Hypogonadotropic hypogonadism ^#^		1 (1.1)	9 (3.2)	0.318 (0.040; 2.543)	0.280	-	-
Premature ovarian insufficiency ^#^		1 (1.1)	4 (1.4)	0.729 (0.080; 6.602)	0.778	-	-
Myoma ^#^		23 (24.2)	35 (12.6)	2.218 (1.232; 3.994)	0.008	2.108 (1.008; 4.409)	0.048
Endometrial polyp ^#^		6 (6.3)	19 (6.8)	0.919 (0.356; 2-374)	0.861	-	-
Male factor ^#^		58 (61.1)	110 (39.6)	2.394 (1.485; 3.859)	<0.001	2.105 (1.156; 3.833)	0.015
Otherwise unexplained infertility ^#^		3 (3.2)	65 (23.4)	0.107 (0.033; 0.349)	<0.001	0.204 (0.057; 0.733)	0.015
Presence of any hydrosalpinx ^#^		9 (9.5)	2 (0.7)	14.442 (3.062; 68.122)	<0.001	13.323 (2.679; 66.253)	0.002

Date are presented as * median (IQR) or ^#^ number (%); Nagelkerkes R^2^ for this model = 0.19.

## Data Availability

The data are available from the corresponding author upon reasonable request.

## Abbreviations

ART	assisted reproductive technologies
BMI	body mass index
FHA	functional hypothalamic amenorrhea
FSH	follicle-stimulating hormone
HSG	hysterosalpingography
HyCoSy	hysterosalpingo-contrast sonography
IVF	in vitro fertilization
PCOS	polycystic ovarian syndrome
POI	premature ovarian insufficiency
IUI	intrauterine insemination
